# Perceived Stigmatization in Patients with Opioid Use Disorder followed up at Arrazi Psychiatric Hospital in Salé

**DOI:** 10.1192/j.eurpsy.2025.922

**Published:** 2025-08-26

**Authors:** N. Ait Bensaid, M. Sabir, F. El Omari

**Affiliations:** 1 arrazi psychiatric hospital, Sale; 2 hopital psychiatrique arrazi salé, Salé; 3 HAS, arrazi psychiatric hospital, Sale, Morocco

## Abstract

**Introduction:**

Stigmatization has been associated with unfavorable outcomes for individuals with mental illnesses, as it acts as a barrier to seeking help and achieving age-appropriate functional goals. Among mental disorders, people with schizophrenia are most frequently stigmatized, followed by those with alcohol-related disorders, substance use disorders, and depressive disorders.

Substance use disorders have often been viewed as stigmatizing conditions

**Objectives:**

To assess perceived stigmatization and its consequences among patients followed and hospitalized for the management of opioid use disorder in the addiction service at Arrazi Psychiatric Hospital in Salé.

**Methods:**

This is a descriptive cross-sectional study using a questionnaire that includes sociodemographic and clinical criteria, as well as an assessment of perceived stigmatization among patients followed and hospitalized for the management of opioid use disorders in the addiction service at Arrazi Psychiatric Hospital in Salé.

Perceived stigmatization was measured using the Perceived Stigma of Addiction Scale (PSAS). This scale consists of 8 items measuring perceived stigma among substance users.

**Inclusion criteria:**

patients of both sexes, aged 18 to 65, diagnosed with opioid use disorder.

**Results:**

A total of 72 responses were retained. Approximately 60% were male, the majority lived with their families, and about 85% were unemployed. Codeine and tramadol were the most commonly used substances, while heroin was less prevalent. Psychiatric comorbidity was present in about the majority of participants (anxiety disorder, depressive disorder, personality disorder, and 2% with psychosis).

The mean (standard deviation) score of the perceived stigma scale for the study population was 21.23. The mean perceived stigma score was significantly associated with the place of residence, with significantly higher perceived stigma found in participants living alone. Stigmatization was also higher depending on the route of opioid administration and was correlated with the participants‘ socio-economic status.

**Conclusions:**

The study suggests that stigma is a major concern among patients with opioid use disorders. Further research is needed to assess the relationship between stigma and the extent of substance use, personality factors, treatment-seeking behaviors, and its impact on quality of life. Studies could evaluate the evolution and trajectory of stigma throughout treatment in order to develop interventions that could help address the issue of stigma in mental health.

**Disclosure of Interest:**

None Declared

